# Autophagy and the Lysosomal System in Cancer

**DOI:** 10.3390/cells10102752

**Published:** 2021-10-14

**Authors:** Suresh Kumar, Miguel Sánchez-Álvarez, Fidel-Nicolás Lolo, Flavia Trionfetti, Raffaele Strippoli, Marco Cordani

**Affiliations:** 1 Autophagy Inflammation and Metabolism Center of Biomedical Research Excellence, University of New Mexico Health Sciences Center, Albuquerque, NM 87131, USA; 2 Department of Molecular Genetics and Microbiology, University of New Mexico Health Sciences Center, Albuquerque, NM 87131, USA; 3 Department of Biological Sciences and Bioengineering, Indian Institute of Technology, Kanpur 208016, India; 4 Mechanoadaptation & Caveolae Biology Laboratory, Cell and Developmental Biology Area, Centro Nacional de Investigaciones Cardiovasculares (CNIC), Melchor Fernández Almagro, 3, 28029 Madrid, Spain; miguel.sanchez@cnic.es (M.S.-Á.); flolo@cnic.es (F.-N.L.); 5 Department of Molecular Medicine, Sapienza University of Rome, Viale Regina Elena 324, 00161 Rome, Italy; flavia.trionfetti@uniroma1.it; 6 National Institute for Infectious Diseases L. Spallanzani, IRCCS, Via Portuense, 292, 00149 Rome, Italy; 7 IMDEA Nanociencia, Faraday 9, 28046 Madrid, Spain; marco.cordani@imdea.org

**Keywords:** TFEB, autophagy, mTOR, AMPK, lysosomes, cancer, nanoparticles

## Abstract

Autophagy and the lysosomal system, together referred to as the autophagolysosomal system, is a cellular quality control network which maintains cellular health and homeostasis by removing cellular waste including protein aggregates, damaged organelles, and invading pathogens. As such, the autophagolysosomal system has roles in a variety of pathophysiological disorders, including cancer, neurological disorders, immune- and inflammation-related diseases, and metabolic alterations, among others. The autophagolysosomal system is controlled by TFEB, a master transcriptional regulator driving the expression of multiple genes, including autophagoly sosomal components. Importantly, Reactive Oxygen Species (ROS) production and control are key aspects of the physiopathological roles of the autophagolysosomal system, and may hold a key for synergistic therapeutic interventions. In this study, we reviewed our current knowledge on the biology and physiopathology of the autophagolysosomal system, and its potential for therapeutic intervention in cancer.

## 1. Introduction

The regulation of autophagy and the dynamics of the lysosomal system are intertwined to ensure cellular health and quality [1,2,3], and their disruption contributes to the physiopathology of several diseases, including cancer, neurodegeneration, metabolic and ageing-related disorders, and inflammatory diseases [1,4,5]. Transcription factor EB (TFEB), one of the four members of the MiTF/TFE3 family [6], is a master transcriptional regulator of both autophagy and lysosomal components [7,8,9,10,11]. In addition, TFEB transcriptionally regulates the expression of genes involved in mitochondrial quality control [12], lipid metabolism [13], and lysosomal exocytosis [14].

Cancer is one of the prime causes of death worldwide [15]. Despite recent advances, this disease still poses a major challenge to public health [15]. Several signaling pathways are frequently altered in cancer [16,17,18], among which autophagy regulatory networks and the lysosomal system represent prominent examples with potential therapeutic implications [19,20,21,22,23,24]. Lysosomotropic drugs such as chloroquine and hydroxychloroquine are currently being tested in the clinic [24,25,26]. Several other drugs, which inhibit lysosomal function, have shown efficacy against different types of cancers [20,24], not only inhibiting lysosomal function, but also disrupting autophagy-dependent processes, as lysosomal damage affects terminal steps of autophagy [23,24]. Several drugs targeting autophagy inhibit proliferation across several cancer-cell types [20,24].

TFEB and related proteins frequently behave as oncoproteins, as they have a key role in the progression of different cancer types [27,28] through the transcriptional control of different processes contributing to tumor-cell survival, metastasis, and chemoresistance [28]. TFEB does not only directly control autophagy and lysosomal dynamics, but also regulates mechanistic Target of Rapamycin (mTOR) [29], a signaling kinase onto which nutrient sensing and anabolic cues converge and which negatively regulates autophagy [30,31,32,33]. In this study, we reviewed the contributions of autophagy and the lysosomal system to cancer progression and chemoresistance, and the roles of TFEB therein.

## 2. Autophagy: An Essential Homeostatic Process

**Figure 1:**
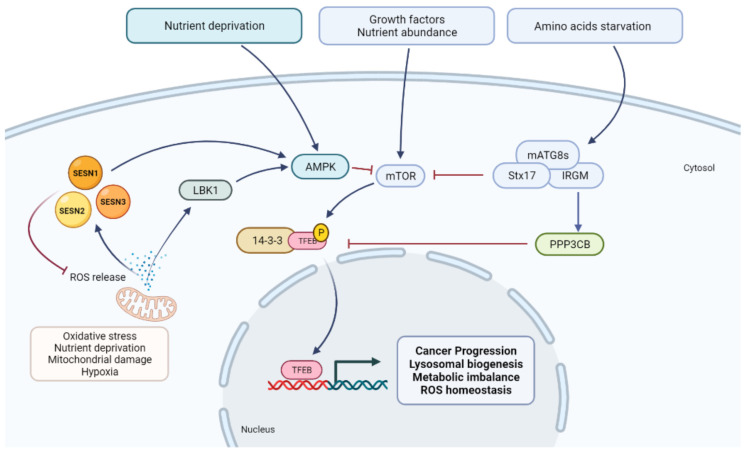
Main extracellular stimuli and intracellular pathways controlling TFEB activation. TFEB is the master transcriptional regulator of both autophagy and lysosomal components, responding to important biological pathways and cellular functions. TFEB translocation to the nucleus depends on its phosphorylation status. Various extracellular and intracellular stimuli including growth factor/nutrient abundance or deprivation and oxidative stress activate, among others, LBK1/AMPK and/or mTOR signaling which control TFEB phosphorylation status. Once phosphorylated, TFEB is sequestered in the cytosol by 14-3-3 proteins. Conversely, during starvation TFEB is dephosphorylated by PPP3CB enabling its nuclear translocation.

Autophagy is a homeostatic process that delivers cell components and structures to lysosomes for degradation and recycling. Autophagy gets rid of cytosolic waste, including damaged organelles and protein aggregates, and contributes to the clearance of invading pathogens. The autophagic machinery is conserved from yeast to mammals [34], and its components control distinct steps to achieve a tight control of this process. Autophagy is initiated by the Unc-51-like autophagy-activating kinase (ULK1) complex, which receives input on cell energy balance, nutrient availability, and growth signaling from mTOR and AMP-activated protein kinase (AMPK) signaling networks [30,31,33,35] (Figure 1). Apart from nutrient availability, viral infections can positively regulate the autophagic process, both directly, through the influence of viral elements on autophagic proteins, and indirectly, through the activation of cellular-stress responses, which, in turn, stimulate autophagy [36,37].

Autophagy-related proteins 2 and 9 (ATG2 and ATG9) provide phospholipids for the nucleation of autophagosome membranes [38,39,40], which are further matured by the ATG14/beclin1/VPS34 complex [41,42]. ULK1 and beclin1 complexes are positively regulated by the cofactor AMBRA1, which is required for their regulative ubiquitination [43,44]. In mammals, the autophagy conjugation machinery then regulates the lipidation of ATG8 proteins (mATG8) [34,45]. Each of the core components of the endosomal sorting complexes required for transport (ESCRT) and machinery (ESCRT complex 0-III) are necessary for the full maturation and sealing of the autophagosome [46,47], onto which syntaxin-17 (STX17), an autophagosomal SNARE protein, is recruited [48] with the assistance of mATG8s and autophagy factor IRGM [49]. STX17 regulates, together with other proteins such as Vesicle-Associated Membrane Protein 8 (VAMP8) and synaptosomal-associated protein 29 (SNAP29), the fusion of the autophagosome with the lysosome [48]. Notably, mammalian STX17 also contributes to the first steps of autophagosome formation downstream of TANK-binding kinase 1 (TBK1) activity, which feeds into the activation of cell defense [50].

**Figure 2:**
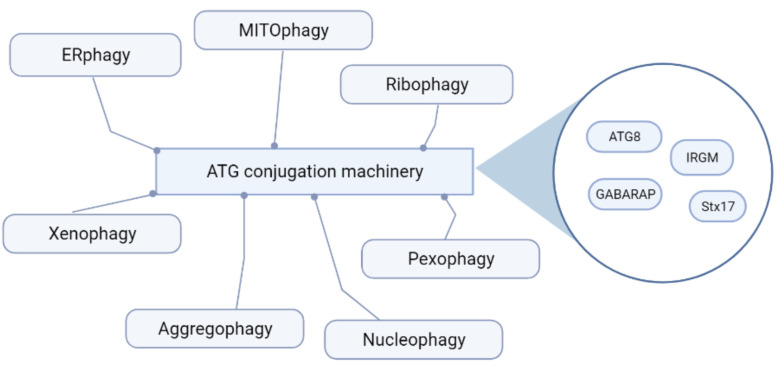
Distinct autophagic routes. Depending on the specific structures and cargoes initiating autophagy, different autophagic routes have been elucidated.

Autophagy leads to cargo degradation irrespective of its identity. However, while core components of the autophagic machinery (i.e., the ATG conjugation machinery) are common to most routes, the autophagy of specific structures and cargoes exhibits particularities in its regulation and the specific source of phagosome membranes: ERphagy (degradation of ER) [51], mitophagy (selective degradation of damaged mitochondria) [52], pexophagy (autophagy of damaged peroxisomes) [53], ribophagy (degradation of ribosomes) [54], nucleophagy (degradation of nuclear membranes) [55,56], xenophagy (degradation of invading pathogens by autophagy) [57,58], or aggrephagy (autophagic clearance of protein aggregates) [59] (Figure 2). Selective cargo autophagy generally requires specific receptors, e.g., selective degradation of ER requires different receptors such as FAM134B [51], CCPG1 [60], RTN3 [61], and TEX264 [62,63]. In contrast, mitophagy requires NDP52, optineurin, and TAXBP1 as receptors [64] and p62 [65]. Certain receptors are common to more than one autophagic route: for example, NDP52 and optineurin control mitophagy [64] as well as xenophagy [66,67]. Route-specific regulators have also been described, apart from cargo-specific receptors. Microautophagy involves invagination of the lysosomal membrane to capture cargo for degradation [68], and involves both components of the autophagic machinery, including the conjugation machinery [69], and sequestosome-like receptors (SLRs) [70]. LC3-associated phagocytosis (LAP) requires the autophagy conjugation machinery but is controlled by RUBICON [71,72,73], which is an inhibitor of conventional autophagy [42,74]. Similar to LAP, other non-canonical autophagy processes utilize ATG conjugation and do not require autophagy-initiation machinery [75,76,77]. While a major share of therapeutic strategies rely on intervening major core components and regulators (the focus of our section below), these relatively recent mechanisms are regarded as interesting future candidates for personalized therapy of specific disorders.

## 3. mTOR Signaling: A Key Regulatory Node Curbing Autophagy

mTOR is a serine/threonine kinase which functions at the interface between nutrient sensing and different cellular processes leading to cell growth and proliferation [78]. Since its discovery, there has been a progressive understanding of the different pathways orchestrated by mTOR, unveiling its role as a central hub for cellular and organismal physiology in all eukaryotes [79].

mTOR is composed by two distinct protein complexes in metazoans, named mTORC1 and mTORC2. Although they share some core protein components, different accessory elements account for structural and functional differences in rapamycin sensitivity and substrate specificity. mTORC1 is mainly constituted by mTOR, the mammalian lethal with SEC13 protein 8 (mLST8) [80], and the regulatory-associated protein of mTOR, RAPTOR [81]. Its major substrates are eukaryotic translation initiation factor 4E-binding protein 1 (4E-BP1) and p70S6 kinase (S6K1), through which mTORC1 controls protein synthesis, nutrient uptake, and autophagy, all leading to positive regulation of cellular growth.

mTORC2 bears, instead of RAPTOR, the rapamycin-insensitive companion of mTOR (RICTOR) protein [82,83], which interacts and binds to MAPK-associated protein 1 (mSIN1) [84,85]. Members of the AGC kinase family (including AKT, SGK, and PKCα) are major mTORC2 substrates, through which it regulates cytoskeletal behavior as well as different pro-survival pathways, all impinging on cellular proliferation.

Although it is expressed in all tissues, from the point of view of its function as a nutrient sensor mTOR is probably most important in metabolically intensive locations, such as muscle, liver, or adipose tissue. After feeding, insulin, secreted by the pancreas, activates mTORC1 and mTORC2, promoting glucose uptake and storage in the form of glycogen in skeletal muscle [86] as well as amino-acid incorporation, leading to muscle growth. In contrast, fasting activates a catabolic program that induces autophagy, leading to protein degradation and liver gluconeogenesis. Several lines of evidence indicate that this balance between anabolism and catabolism is fundamentally regulated by mTOR [87,88]. Liver-specific RICTOR-knockout mice, for instance, show alterations in lipid storage and hyperglycemia as a result of systemic insulin resistance [89,90,91], indicating that mTOR signaling alterations contribute to the development of diabetes. Similarly, adipose-specific RAPTOR-knockout mice are resistant to diet-induced obesity and present low body weight [92,93], in accordance with the role of mTOR in lipid anabolism and adipose-tissue function, and whole-body homeostasis therein [94,95].

The dysregulation of mTOR signaling is frequent in tumors [96]. Aberrant activation of mTORC1 signaling, for instance, favors tumor growth by eliciting cancer cells to bypass metabolic checkpoints. Hyperactivation of mTORC2 signaling, on the other hand, boosts metastasis by supporting AKT-dependent cytoskeletal remodeling [97]. Despite its complexity, the role of mTOR in cancer has led to different therapeutic strategies, including ‘rapalogs’—rapamacyin derivatives—which have shown efficacy in certain contexts [98]. Prolonged mTOR inhibition can however lead to reactivation of cancer growth [99,100], stressing the need for further research to better understand the multifaceted impact of mTOR signaling on cancer progression.

## 4. Transcriptional Control of the Autophagolysosomal Machinery: TFEB and Its Regulation

**Figure 3:**
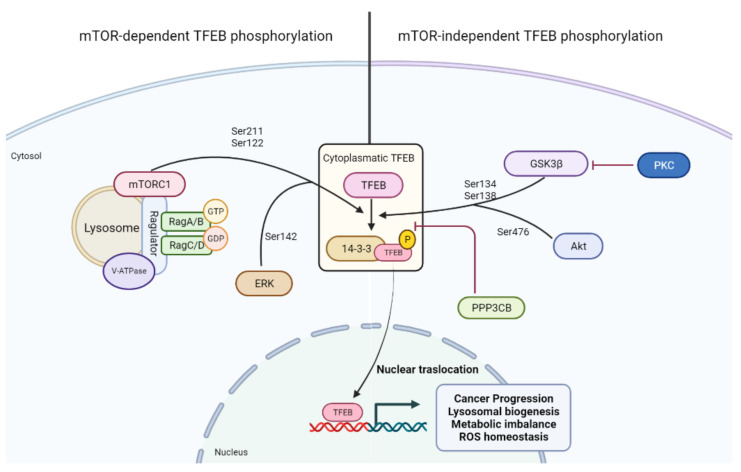
Regulation of TFEB activation by specific phosphorylations.

TFEB is a major transcription factor which regulates the transcription of genes involved in several biological pathways, and participates in important cellular functions, including autophagy [7], lysosomal biogenesis [101], lysosomal exocytosis [14], lipid metabolism [13], mitophagy [102], and mitochondrial biogenesis [12]. A major regulatory layer controlling TFEB nuclear translocation and activity is affected through phosphorylation, mainly controlled by the mTOR kinase [103,104], and by a phosphatase, PPP3CB [105] (Figure 1). mTOR phosphorylates the Ser211 residue of TFEB [104], eliciting its interaction with 14-3-3 proteins which sequester TFEB in the cytosol [104,105] (Figure 3). 14-3-3 proteins also interact with other members of the MiTF family [104,106,107]. Notably, TFEB interacts with mTORC1 but not with mTORC2, and its localization to lysosomes, cytosol, or the nucleus depends on the activation state of Rag GTPases [29,103,108]. Conversely, TFEB modulates the lysosomal localization and function of mTORC1 [29]. Besides the Ser211 residue, mTOR also phosphorylates TFEB at Ser122 [109].

TFEB can be phosphorylated by several kinases either dependent or independent of mTOR. Notably, only the mTORC1 complex can regulate TFEB activity through the phosphorylation of Ser122 and Ser211. Other kinases involved in the mTOR pathway may interact with TFEB. In particular, ERK1 determines Ser142 phosphorylation and subsequent TFEB cytoplasmic sequestration. GSK3β and Akt may regulate TFEB nuclear translocation in an mTOR-independent manner, phosphorylating Ser134–138 and Ser476, respectively.

There are additional kinases which phosphorylate TFEB at other residues, contributing to the regulation of its nuclear translocation [110]. Indeed, TFEB is phosphorylated by ERK at Ser-142 residue, also blocking its nuclear translocation [101]. In contrast, TFEB phosphorylation at Ser138 controls its nuclear export [110,111].

Other kinases independent from mTOR have been implicated in TFEB nuclear translocation and in the regulation of the lysosome system [110]. PKC, for instance, controls nuclear translocation of TFEB in an mTOR-independent manner [112]; GSK3β phosphorylates TFEB at Ser134 and Ser138, which, like mTOR phosphorylation, keeps TFEB in the cytosol [112]; AKT phosphorylates TFEB at its Ser467 residue, thus blocking its translocation to the nucleus [113].

TFEB is dephosphorylated by a calcineurin phosphatase, PPP3CB, during starvation. PPP3CB dephosphorylates phosphor-Ser211 TFEB [104], releasing it from 14-3-3 proteins and eliciting its nuclear translocation [105]. The relevance of TFEB for cell homeostasis is further highlighted by the increasing number of additional posttranslational modifications recently described (acetylation, SUMOylation) [114,115], reflecting the integration of several inputs feeding on this central node regulating autophagy and lysosomal function. Finally, TFEB expression is sensitive to different cues challenging cell homeostasis; examples are ER stress, which can promote TFEB upregulation to engage autophagy and ensure lysosomal function downstream with the Unfolded Protein Response (UPR) effectors XBP1 and PERK/ATF4 [116,117,118], and oxidative stress, which induces TFEB nuclear translocation in an NRF2-dependent manner [117].

## 5. TFEB and Autophagy

Current models propose that TFEB operates upstream of the autophagy pathway [1,7,13,119]. TFEB positively correlates with gene expression changes in autophagy genes and relative lipidation of the autophagy marker LC3 [7]. TFEB controls autophagy during different stresses including starvation [7], lysosomal damage [120], neuronal toxicity [121,122] inflammation [123,124,125,126], and infection with pathogens [127,128,129,130]. Recent evidence suggests that TFEB and TFE3 control ERphagy [131] by regulating the expression of the ERphagy receptor FAM134B [51].

Notably, recent reports indicate that autophagy may in turn operate upstream of TFEB to control its nuclear translocation during bulk and selective autophagy [102,132]. Mammalian ATG8 proteins (mATG8s), which are involved in autophagosome elongation [133,134] and autophagosome–lysosome fusion [133,135], also participate in lysosomal biogenesis [136]. mATG8s form complexes with autophagy factor IRGM and SNARE protein Stx17 [49]. Like mATG8s, Stx17 [48,50] and IRGM [137,138] participate in different steps of autophagy. This complex consisting of IRGM, Stx17, and mATG8s [49] controls TFEB nuclear translocation in response to starvation. IRGM and GABARAP (a member of mATG8s family) directly interact with TFEB. IRGM, Stx17, and mAtg8s proteins influence TFEB nuclear translocation by inhibiting mTOR activity in response to amino-acid starvation [132]. mATG8 proteins also control TFEB action at transcriptional level [132]. While mTOR-dependent TFEB phosphorylation leads to TFEB cytosolic retention [103,104,105], IRGM-dependent dephosphorylation favors its nuclear translocation [132]. Therefore, there is a positive feedback loop between autophagy and the lysosomal system to regulate cellular homeostasis.

## 6. TFEB in Lysosomal Biogenesis and Function

Lysosomes are crucial components of the cellular degradation and recycling system. Lysosomes contain approximately 60 different soluble hydrolytic enzymes, which are directly involved in the degradation of macromolecules in other cellular wastes [11]. TFEB is a master regulator of lysosomal biogenesis [101,119,139]. TFEB transcriptionally regulates the gene expression of the CLEAR (coordinated lysosomal expression and regulation) network, the expression of target genes bearing the CLEAR motif, thereby modulating autophagy and lysosomal biogenesis [119,139]. TFEB not only controls lysosomal biogenesis but also other processes associated with lysosomal function such as autophagy [7], endocytosis [140], and lysosomal exocytosis [14]. An additional role for the lysosomal system pertains to the link between membrane trafficking, ER architecture, and mTORC1 activation status [141,142,143]; the tight control of such a central node for lysosomal regulation by mTORC1 reflects the functional coupling of these cellular systems.

## 7. The Autophagolysosomal System and Cellular ROS Homeostasis

The term “Reactive Oxygen Species” (ROS) is used for a heterogeneous group of highly reactive chemical entities containing molecular oxygen—including oxygen radicals (i.e., superoxide (O_2_•−), and hydroxyl (•OH), peroxyl (RO_2_•), and alkoxyl (RO•) radicals), and non-radicals (i.e., hypochlorous acid (HCIO), singlet oxygen (1O_2_), and hydrogen peroxide (H_2_O_2_). Most, if not all of them, are typically by-products of cell metabolism, even under physiological conditions [144,145], although different external agents such as xenotoxins or ionizing radiations can provoke extensive oxidative stress and ROS accumulation [146,147]. Cells have evolved intricate antioxidant systems to curb damaging rises in ROS levels, such as glutathione pair (GSSG/GSH), nicotinamide adenine dinucleotide pair (NADH/NAD+), superoxide dismutases (SODs), catalase, glutathione peroxidases (GPXs), peroxiredoxins (PRXs), or thioredoxins (TRXs) [148,149,150]. These are integrated in different stress responses (UPR, electrophilic-stress response, integrated-stress response, AMPK network) [151,152,153,154,155], triggered by stimuli (nutrient deficiency, metabolic imbalance, lipotoxicity, and proteotoxicity) that can potentially boost ROS accumulation.

Of note, autophagy is commonly considered as an additional branch of these stress networks, and is activated by many of these adverse conditions both through direct links, as well as though the general integration of the mTOR signaling network with these stress pathways [156,157]. Autophagy is an important contributor to cell survival from ROS-inducing stress, by curbing the accumulation of damaged structures and removing faulty organelles acting as sources of ROS [158].

The relevance of this link between ROS production and autophagy is exemplified by the fact that elevated ROS species and/or compromised antioxidant responses are frequent hallmarks of the altered metabolism and environment of tumor cells, often actively promoting tumorigenesis [159,160,161]. These features are both considered appealing therapeutic targets *per se*, and opportunities for synergistic interventions. Two emerging, related therapeutic strategies based on these phenotypic alterations of tumor cells are the use of ascorbate (for which certain tumor cells, such as glioblastomas, exhibit paradoxical differential toxicity through oxidative damage) [162,163] and other strategies leveraging on mechanisms driving ferroptosis, a specific cell-death program triggered by iron-dependent accumulation of peroxidized lipid species [164,165,166]. Autophagy can frequently act as a pro-survival response counteracting these damaging stimuli in different types of tumors [167,168]. However, autophagy itself can be both positively or negatively modulated by these forms of oxidative stress, and may serve as part of the effector mechanism of the ferroptotic cascade [162,169,170,171,172]. Further research is thus warranted to understand the architecture of the underlying networks and the principles of their functioning.

## 8. Modulating the Autophagolysosomal System in Cancer: Therapeutic Opportunities

Autophagy induction by cancer-associated stimuli (oxidative stress, suboptimal nutrient supply, and hypoxia), and its tight relationship with pro-survival cell pathways, support a direct role of autophagy in cancer transformation. However, the role of autophagy in cancer is highly contextual. Autophagy can act both as a tumor suppressor mechanism, favoring the elimination of damaged proteins or organelles, or as tumorigenic, providing a source of nutrients and energy to tumor cells and further favoring their transformation.

Murine models demonstrate that autophagic gene deficiency favors tumorigenesis, at least at initial stages [173]. Deficiency of autophagic genes such as Beclin1 or Atg5 has been found in various cancers, including hepatocellular carcinoma (HCC), breast, ovarian, and prostate cancer [173,174]. Impaired autophagy can promote a tumorigenic environment through ROS dysregulation and chronic induction of inflammatory states [175]. Autophagy defects in mice cause accumulation of p62 aggregates, oxidative stress, and p62-dependent hepatocyte cell death favoring hepatocarcinoma progression [176,177]. In breast cancer, aberrantly expressed p62 may favor the generation of breast stem cells (CSCs) through the induction of MYC oncogene [178].

On the other hand, at advanced cancer stages, increased autophagy can sustain tumor cell growth in the nutrient-deficient, hypoxic tumor microenvironment, and favor chemoresistance by counteracting the damage of cell structures [179]. Further, autophagy promotes resistance to anoikis (a form of cell death induced by cellular detachment from the extracellular matrix) in gliomas, enabling tumor spreading and metastasis [180,181]. However, autophagy inhibition can also favor tumor cell invasiveness through the induction of dedifferentiated, basal phenotypes in breast cancer [182]. Upregulation of autophagy induction confers chemoresistance [20,179,183] and promotes the maintenance and survival of CSCs in different cancers including breast, pancreas, liver, ovarian cancer, osteosarcoma, and glioblastoma [184].

Cancer cells generally grow faster than non-transformed counterparts and have high metabolic demands, so they may use autophagy and the lysosomal pathway to meet high demands for energy and anabolic flux [28]. In fact, similar cancers bearing different genetic mutations may vary for their dependence on autophagy. For instance, tumors with mutations in the RAS–MAPK pathway, such as central nervous system (CNS) tumors bearing a BRAF V600E mutation, but not their wild-type BRAF-expressing counterparts, were found to be strongly dependent on autophagy [19,185]. This discovery paves the way to the translational employ of autophagy inhibition in combination with other therapeutic strategies.

Due to the relevance of autophagy and the lysosome system in cancer biology, their modulation by drugs is a current target in cancer therapy [186,187].

To this purpose, multiple steps in autophagy are currently being considered. Inhibition of ULK1 sensitizes cancer cell to nutrient stress [188] and mTOR inhibitors [189]; inhibition of VSP34 has shown to improve the effect of mTOR inhibition and tyrosine-kinase inhibitor on suppression of cancer growth [190,191]; inhibition of ATG4B, a protease that controls lipidation and delipidation of mATG8s [192], also suppresses cancer progression [193]. Chloroquine and its derivative hydroxychloroquine are lysosomotropic agents which inhibit fusion of autophagosomes with lysosomes [194], and are at different stages of clinical trials against different types of cancers [20,24].

Lysosomes are nutrient-sensing organelles. Lysosomes and their related biological functions, such as endocytosis, phagocytosis, and micropinocytosis, are involved in maintaining energetics in cancer [22]. Lysosomal volume and subcellular localization are changed during cancerous transformation [195]. Lysosomal hydrolases such as cathepsin are upregulated and display altered localization in cancer. Increased cathepsin expression is correlated with cancer progression [196,197]. Lysosomal membrane protein LAMP1 is associated with cancer development and progression [22,198]. Lysosomal V-ATPase has been shown to affect tumor microenvironment [199].

Due to its prominent role as an upstream regulator of autophagy and lysosomal function, TFEB might constitute a priority target for the efficient therapeutic intervention of these routes. RNA-based therapeutics are expected to soar after the success of RNA-based vaccines; in this sense, numerous studies indicate TFEB is an effective target for the modulation of autophagy and lysosomal activity to successfully counteract different pathological conditions, including cancer [200,201,202,203,204]. Reflecting the highly contextual role of autophagy in cancer, while TFEB and related factors have frequently been regarded as oncogenes, TFEB can behave as a tumor suppressor, as recently reported for acute myeloid leukemia (AML) [205]. It must be noted that effective reversion of certain pathological conditions through TFEB modulation may require the simultaneous intervention of associated gene-expression networks, such as those controlled by YAP [206]. Notably, small compounds amenable for human therapeutics such as genistein, 20-deoxygenol, curcumin, or betulinic acid have been reported to be capable of enhancing TFEB-dependent lysosomal activity [207,208,209,210]; other TFEB-modulating compounds were identified in phenotypic screens in *Caenorhabditis elegans* [211]. The synergistic potential of these compounds with other treatments sensitizing tumor cells to autophagic modulation deserves further exploration.

## 9. Nanomedicine May Increase the Potential of Drugs Modulating Autophagy

As described in the previous section, autophagy may play a dual role in cancer depending on cell type and stage, potentially acting both as tumor suppressor and as a promoter of tumor progression [212]. For this reason, both the inhibition of autophagy and its overstimulation are strategies under assessment to counteract cancer, and several drugs, such as hydroxychloroquine (HCQ), 3-methyl-adenine (3-MA), and everolimus, have been approved by the Food and Drug Administration (FDA) and are currently employed in clinics in combination with other chemotherapeutic regimens [179,213].

However, these treatments present a variety of adverse effects such as low specificity, irregular distribution in the body, and rapid drug clearance [214]. For this reason, novel approaches aimed at modulating autophagy are warranted.

Recent advances in nanotechnology offer many tools to counteract cancer with innovative and smart therapeutic agents by overcoming obstacles frequently encountered with standard chemotherapeutics. Novel smart nanomaterials have been engineered that, depending on their chemical–physical proprieties, can be divided into various categories, such as liposomes, polymers, metals, and metal-oxide nanoparticles (NPs) [215]. Most of these nanomaterials are used as nanocarriers to deliver therapeutic molecules such as drugs, proteins, or nucleic acids into specific target sites without affecting healthy tissues [216,217]. In this regard, it must be noted that a major advantage of such an approach consists in the fact that nanocarriers can accumulate in the leaky tumor vasculature, a process known as enhanced permeability and retention effect (EPR). This capability is essential in guaranteeing specificity of the therapeutic system and for its applications in vivo [218]. Moreover, nanocarriers can release their therapeutic cargo in a stable and controlled manner. A plethora of stimuli, such as changes in pH, redox, temperature, or magnetic forces, can trigger the release of drugs by evoking a change in the structures of the nanocomplex, to ensure toxicity exclusively into target tissue, without affecting healthy tissues [219].

Combination therapy with cisplatin and chloroquine in micelles formed by self-assembling hybrid dendritic-linear-dendritic block copolymers (HDLDBCs) increased cytotoxicity in tumor cells while maintaining a low degree of cytotoxicity against non-tumor cells [220]. Lys-05, an autophagy inhibitor which accumulates within and deacidifies the lysosome [221], was hybridized with a lysosomotropic detergent (MSDH) to produce nanoassemblies. The resulting nanoparticles were demonstrated to have excellent pharmacokinetic and toxicological profiles and a dramatic efficacy against tumors in vivo [222].

The surface of gold nanoparticles (Au-NPs) can be easily functionalized with chemotherapeutics or nucleic acids, such as snake-venom-protein toxin NKCT1, monoclonal antibodies, or quercetin, making them excellent autophagy inductors for cancer therapy [223,224,225].

Besides acting as nano-carriers, nanoparticles of specific materials may have the intrinsic ability of altering the complex network of signaling pathways and molecules involved in autophagy regulation, and thus represent an exciting therapeutic approach against different human tumors [226]. Bare iron-oxide NPs are significantly cytotoxic to human lung carcinoma cells (A549 cells), causing ROS-induced autophagy and subsequent cell death, but not to normal human-lung fibroblast cells [227]. Chiral nanomaterials are being developed to modulate autophagy activity in tumors [228], and chiral polymer-modified nanoparticles may induce autophagy-mediated tumor suppression in vivo [229]. Moreover, D- and L-cysteine-modified Cu_2−x_S nanocrystals (NCs) were reported to produce large amounts of ROS in tumor cells, promoting cellular autophagy [230].

The use of modified NPs to intervene in autophagy is not limited to cancer, and many other diseases can be treated by this approach. Indeed, defective clearance of misfolded proteins and/or damaged organelles occurs in a plethora of human diseases, such as muscular or neurodegenerative diseases, and the pharmacological modulation of this process may represent a valid therapeutic approach. For example, cerium oxide nanoparticles (CeO_2_-NPs) were reported to activate autophagy and promote clearance of autophagic cargo, thus exerting neuroprotection. [231]. Furthermore, europium hydroxide nanoparticles [(EuIII(OH)3)-NPs] have been shown to stimulate autophagy flux, reducing mutant-huntingtin-protein aggregation [232].

However, despite such promising potential, the autophagy induction activity of these nanomaterials can also lead to cardiovascular, respiratory, and immune-system toxicity [233]. Hence, the use of nanomedicines in autophagy modulation is at its infancy and the clinical translation of the results thus far obtained is still a challenge [234].

In conclusion, further effort is needed to understand the molecular mechanisms and principles governing the autophagolysosomal system, for its efficient, safe, and personalized intervention across multiple diseases, including cancer.

## Data Availability

Not applicable.
